# The Contribution of the Elastic Reaction is Severely Underestimated in Studies on Myofibril Contraction

**DOI:** 10.3390/ijms10030942

**Published:** 2009-03-02

**Authors:** Enrico Grazi, Sara Pozzati

**Affiliations:** Dipartimento di Biochimica e Biologia Molecolare, Università di Ferrara, via Borsari 46, 4410 Ferrara, Italy; E-Mail: pzzsra@unife.it

**Keywords:** Huxley, Simmons, manoeuvre, elastic reaction, myofibrils contraction

## Abstract

We have considered the Huxley-Simmons manoeuvre. On the assumption that the quick release is an elastic process and on the basis of the isometric tension and of the stiffness of the muscle fibre we calculated that the spontaneous release of the fibre requires ∼43 μs, which is much faster than the observed release, ∼180 μs. We concluded that the observed quick release is a guided process. After proper selection of the mass and of the stiffness of the system we mimicked the early recovery and noticed that most of the energy required to accomplish the early recovery is supplied by the kinetic energy accumulated during the course of the quick release. We computed that the frequency of the working strokes in the half sarcomere was between 4×10^6^ and 40×10^6^ s^−1^. This is not to say that the ATPase rate constants are accumulative but only that the overall frequency of the working strokes in the half saromere is many orders of magnitude faster than the average ATPase rate constant. With this frequency no part of the Huxley-Simmons manoeuvre, quick release included, escapes the control of the working stroke. This means also that there is no reason to take the early recovery as an indication of the length of the working stroke.

## 1. Introduction

The Huxley-Simmons manoeuvre consists into applying a small, very rapid, length change to a single muscle fibre in the isometric state (quick release) and to measure the subsequent rapid tension recovery at fixed sarcomere length [1]. The mechanics of the manoeuvre were explained by assuming the cross-bridge to be composed by two structural elements, an elastic one and a visco-elastic one capable of readjusting the tension of the system. The system was then improved by introducing an additional elastic element [2]. The Huxley-Simmons manoeuvre became suddenly very popular because 1) quick release was considered to synchronize the cross-bridges and 2) rapid tension recovery was taken as a manifestation of the working stroke, even though the event occurs at fixed fibre length.

The Huxley-Simmons manoeuvre was utilized to investigate: a) the rapid regeneration of the myosin power stroke in contracting muscle [3,4]; b) the structures involved in the Huxley-Simmons manoeuvre [1]; c) the Huxley-Simmons manoeuvre and the 14.5 nm X ray reflection [5]; d) elastic bending and active tilting of myosin heads during muscle contraction [6]; e) the quick release and the frequency of the power strokes [7,8]. In our opinion some of the results of these experiments were overinterpreted and alternative interpretations can be offered. Careful analysis of the work of Ford *et al*. [2] shows that their quick release is not a simple elastic phenomenon, but involves rather the contribution of at least an additional force that opposes the shortening. The study of the behaviour of an elastic body reflected by a hard boundary teaches how to mimic the rapid force recovery without fibre lengthening and cast doubt on the hypothesis that the early force recovery represents the power stroke. Full consideration of the contribution of the elastic energy made available by the quick release questions the proposal of Lombardi *et al*. [4], that the stroking rate is more than 10 fold higher than the ATPase rate.

## 2. Results

### 2.1. Mimicking the quick release

In this section we show that, in the work of Ford *et al*. [2], the quick release is not purely elastic but is a complex event determined, in addition to the elastic force, by at least one extra force that opposes the shortening.

**Figure 1.**
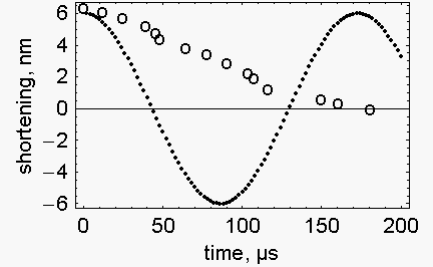
*Time course of the quick release* – Observed change of length during a quick release of 6 nm/half sarcomere (open circles), taken from Figure 14 of Ford *et al*. [2]. Calculated, spontaneous change of length during the same release (filled circles). The conditions used for the calculation were those of Figure 14 of Ford *et al*. [2]: P_0_, = 270 kN/m^2^; fibre length, 5.34×10^−3^ m; fibbre section, 1.89×10^−8^ m^2^; fibre density (corrected for the markers), 1.19×10^3^ Kg/m^3^; sarcomere length, 2×10^−6^ m; fibre stiffness, 0.015927 N/m.

The quick release of a muscle fibre in the isometric state is considered an elastic event. Accordingly we calculated the rate of shortening as well as the force of an elastic fiber during a spontaneous release (Appendix A). Figure 1 presents the calculated (filled circles) and the experimental (open circles) shortening of a muscle fibre during a quick release of 6 nm per half sarcomere (data from Figure 14 of [2]). It is evident that the calculated release is faster (∼43 μs) than the experimental one (∼180 μs).

**Figure 2.**
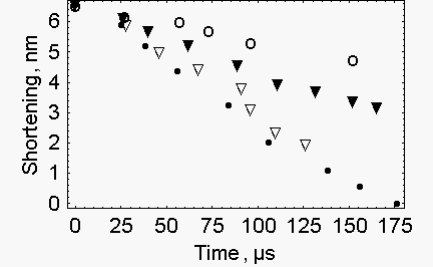
*Measured changes of length during the quick release* - data are taken from Ford *et al*. [2]. The releases are given in nm per half sarcomere: 1.5 nm (open circles); 3.0 nm (filled triangles); 4.5 nm (open triangles); 6.0 nm (filled circles).

Furthermore the rate of the experimental shortening is slow at the beginning, faster in the middle and slow again at the end. This final slowing clearly requires an extra force that opposes the shortening. This means that the quick release is a guided event, in fact different rates of shortening are recorded for releases of different length (Figure 14 of [2]), (Figure 2). This cannot occur in a spontaneous elastic release since all the releases start at the same isometric force.

**Figure 3.**
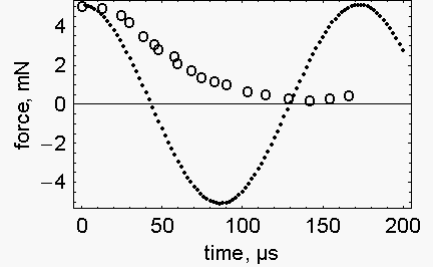
*Time course of the force during a quick release* – Observed change of force during a quick release of 6 nm/half sarcomere (open circles). Calculated change of force during the same release (filled circles). The conditions used for the calculation were those of Figure 14 of Ford *et al*. [2]: P_0_, = 270 kN/m^2^; fibre length, 5.34×10^−3^ m; fibre section, 1.89×10^−8^ m^2^; fibre density (corrected for the markers), 1.19×10^3^ Kg/m^3^; sarcomere length, 2×10^−6^ m; fibre stiffness, 0.015927 N/m.

Figure 3 presents the calculated (filled circles) and the experimental (open circles) change of force associated with the quick release of 6 nm per half sarcomere. Again the decrease of force is faster in the calculated spontaneous release than in the measured one.

### 2.2. The rapid recovery of an elastic body

In this section we show that an elastic body is reflected by a hard boundary because of the kinetic energy accumulated during the compression (shortening) phase.

**Figure 4.**
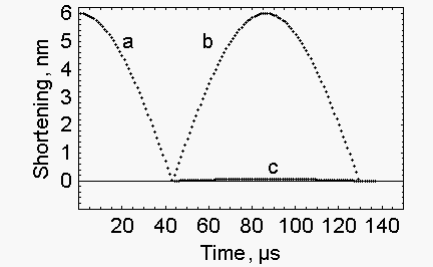
*Quick release and subsequent lengthening due to the reflection against a hard boundary –* a. Quick release, the conditions are the same as for Figure 1, calculations are made as described in Appendix A. b. Rapid recovery, the conditions are the same as for Figure 1, initial kinetic energy, 0.0815917 μJ; fibre stiffness, 0.015927 N/m; fibre mass, 1.2×10^−7^ Kg; calculations are made as described in Appendix B. c. “Isometric” rapid recovery, initial kinetic energy, 0.0815917 μJ, fibre stiffness, 1.2713×10^6^ N/m; fibre mass, 9.608×10^−4^ Kg; calculations are performed as described in Appendix C.

**Figure 5.**
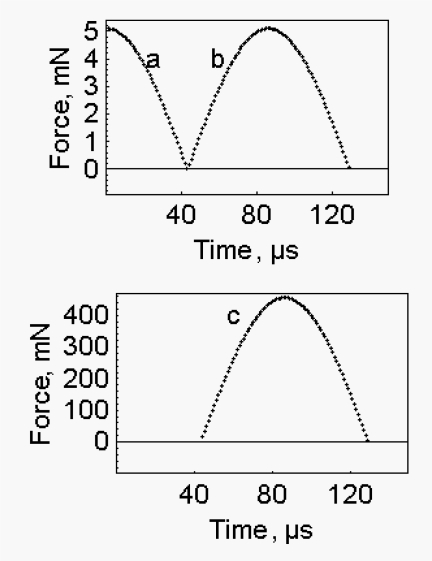
*Change of the elastic force during the shortening accompanying the quick release and the subsequent lengthening* - The conditions are as described in Figure 4. a. Quick release. b. Rapid recovery. c. “Isometric” rapid recovery.

If the quick release of a muscle fibre in the isometric state is an elastic event [2] the energy for the rapid recovery is, at least partially, provided by the quick release itself. In the case of an elastic body the reflection against a hard boundary generates the behaviour shown in Figure 4, where the shortening due to quick release (Figure 4a) is followed by the lengthening due to the reflection against the hard boundary (Figure 4b). The reflection is obtained at the expense of the kinetic energy accumulated during the quick release (Figure 5a), the kinetic energy is converted into potential energy and the elastic force is restored (Figure 5b). Calculations were performed as described in Appendix B.

### 2.3. The rapid recovery of an elastic body at constant length

In this section we mimic the rapid force recovery that occurs without change of fibre length. We show that the simulation requires to increase suitably the stiffness and the mass of the system. In the experiments performed by Ford *et al*. [2] at the end of the quick release the change of fibre length is forbidden and the force recovers at constant length. This situation is mimicked in the elastic body by the procedure described in Appendix C. In short a stressed elastic body is released, as a result kinetic energy accumulates. 1) This kinetic energy is utilized for the rapid recovery; 2) the change of length of the elastic body is minimized by increasing the stiffness of the system (Figure 4c); 3) the time of the recovery of the force is increased by increasing the mass of the body. Since in the transition between quick release and rapid recovery energy conservation is assumed and since stiffness increases a huge increase of the elastic force takes place (Figure 5c). The expected maximum force is 0.45553 N and is given by the product of the virtual fibre stiffness, 1.2713×10^6^ N/m, time the calculated increase of the length of the half sarcomere, 6.71×10^−11^ m, time 5340, where 5340 is the number of the half sarcomeres in the fibre.

### 2.4. Mimicking the rapid recovery in muscle fiber

The rapid force recovery takes place at constant length. This behaviour can only be explained by a stop imposed by the measuring device to fibre lengthening. We show here that the stop is modeled by the suitable increase of the stiffness and of the mass of the system.

**Figure 6.**
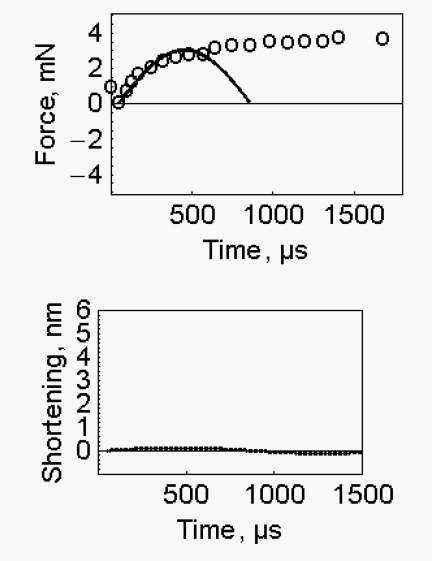
*Mimicking the quick recovery*- Upper part of the figure, open circles: experimental force recovery after a quick release of 6 nm per half sarcomere, data are taken from Figure 14 of Ford *et al*. [2]. Upper part of the figure, filled circles: wave obtained by reflection of the spontaneous quick release, fibre stiffness 1.27×10^6^ N/m, fibre mass 0.9608×10^−3^ Kg, kinetic energy 97 nJ. Lower part of the figure: the recovery of the fibre length display a wave form of small amplitude.

In the experimental rapid recovery of muscle fibre, a large fraction of the original isometric force is recovered while the original length of the fibre is not. To model this dissociation the putative stiffness and the putative mass of the fibre were increased and a suitable value for the kinetic energy was selected. At the end of the spontaneous quick release (Figure 1, filled circles) the fiber mass, corrected for the markers, is 1.2×10^−7^ Kg; the rate of fibre shortening is 1.16564 m/s; the kinetic energy, at zero force, is 81.6 nJ. At the end of the experimental quick release of 6 nm per half sarcomere (Figure 14 of Ford *et al*. [2]), only 0.97 nJ are available. This shows that the kinetic energy made available in the course of the spontaneous quick release (81.6 nJ) was largely dissipated in the experimental procedure. Under these conditions, to mimic the initial part of the experimental rapid recovery after a quick release of 6 nm per half sarcomere (Figure 14 of Ford *et al*., [2]) fibre stiffness was raised to 4760 N/m and fibre mass to 0.32427×10^−3^ Kg. If these figures appear unreasonably high, attention should be paid to the change of setting of the measuring equipment (that allows the sudden stop of the length change of the fibre) and to its equivalence in change of mass and stiffness (hoping that these are known). The simulated rapid recovery (Figure 6 upper part, filled circles) mimics the experimental one. The associated small change of fibre length is shown in the lower part of Figure 6.

### 2.5. Rapid regeneration of the myosin power stroke in contracting muscle

We show here that the proposal of Lombardi *et al*. [4], that the stroking rate is more than 10 fold higher than the ATPase rate, is questionable because the contribution of the elastic energy was ignored. Lombardi *et al*. [4] imposed a sequence of four quick releases of 4.43 nm per half sarcomere at the plateau of an isometric tetanus at intervals of 8 ms. They showed that tension before the steps reduced progressively until a steady value was attained with the third step (Figure 3 and Table 2 of Lombardi *et al*. [4]. Furthermore they assumed a stroking rate per myosin head of 77 s^−1^. Since the estimated rate of ATP splitting per myosin head is ∼6 s^−1^ [9,10] the conclusion of the authors was that the stroking rate is more than 10 fold higher than ATPase rate. The authors overlook the contribution of the kinetic energy made available by each quick release. Energy that becomes even larger in more recent experiments where the step changes of length are quicker (∼0.1 ms) [11]. Under these circumstances it does not appear justified to relate the frequency of the quick releases to the ATPase rate and to the frequency of the power stroke.

### 2.6. Structures involved in the Huxley-Simmons manoeuvre

In this section we propose that all the components of the attached cross-bridge and not only one elastic element, participate to the quick release. Quick release and rapid recovery are the expression of the activity of a single or of more physical structures? Following the proposal of H.E. Huxley [12] that the force-generating structure of the cross-bridge can attach itself to the actin filament in a constant configuration, Huxley and Simmons [1] specifically associated the quick release to one elastic element and the rapid recovery to an additional, visco-elastic element in series with the former.

The quick release is a sequence of events that involve the actin filament, the force-generating structure of the cross-bridge, a 40 nm linkage and, finally, the backbone of the thick filament. Since the force-generating structure of the cross-bridge and the putative 40 nm linkage are in series the associated force must fade concomitantly with the quick release. Furthermore, as shortening readjustment proceeds, the change in length must be shared by the two components of the cross-bridge, in reason of their respective stiffness. There is in fact no reason to associate the quick release to the elastic element only. On the contrary both the structural elements must participate to the quick release.

### 2.7. The Huxley-Simmons manoeuvre and the 14.5 nm X ray reflection

By studying the rapid stretching of muscle fibres in the isometric state Lombardi *et al*. [5] found that, during the length step (and the force increase), the intensity of the 14.5 nm X-ray reflection significantly decreased thus showing that a significant part of the instantaneous elasticity of muscle resides within the myosin head. This contradicts the observation that the 14.5 nm X-ray reflection apparently does not change during the elastic response to 6 nm shortening step [3]. The discrepancy was explained by Lombardi *et al*. [5] by assuming that the long axis of the myosin head were offset from the perpendicular to the filament axis in the isometric state and that, during the shortening step, the heads would move through the perpendicular, with no effect on the distribution of the mass along the filament or the intensity of the 14.5 nm X-reflection. It is thus clear that both in release and in stretching the instantaneous elasticity is accompanied by tilting of the cross-bridges and that the elastic element and the force generating element are likely to merge in a single structure.

## 3. Discussion

### 3.1. About the energy associated to the elastic component of the Huxley-Simmons manoeuvre

If the fast component of the Huxley-Simmons manoeuvre is elastic, the question is then how the elastic energy associated with the rapid shortening is reallocated. To answer the question we modelled an elastic system. The operations had not the aim of mimicing muscle contraction, a much more complex phenomenon, but only to dissect the energy conversions taking place in the course of the rapid release. In doing so we found that a significant amount of elastic energy was made available to further events such as the quick recovery. This observation certainly changes the overall economy of the Huxley-Simmons manoeuvre.

### 3.2. The relationship between the rate of the quick release and the rate of the early recovery

If part of the early recovery is generated by the kinetic energy accumulated in the course of the quick release, a relationship should be found between the rates of the two phenomena. This relation is traced in experiments where the length changes are complete in a fixed time therefore the rate of shortening (and the kinetic energy) increase with the length of the step. In these experiments the rates of the tension transients of the early recovery increase with the step size [11,13,14]. A feature that was never recognized. Furthermore, while formulating their cross-bridge model, Piazzesi and Lombardi [13] assumed the step-length change to be complete in zero time while, in fact, it was complete in 120 μs. This “simplification” would have never been proposed if the authors had appreciated the importance of the kinetic energy of the quick release in the economy of the Huxley-Simmons manoeuvre.

### 3.3. Cross-bridges synchronization

In the isometric state, near the beginning of the working stroke, the actin-attached cross-bridges have their lever arm angles spread over a relatively narrow range, ±20–25° on either side of a mean orientation that is about 60° away from their orientation at the end of the working stroke [15].

During quick releases, the dispersion of the cross-bridges remains approximately constant and the bunched distribution of attached heads is shifted further and further through the working stroke, as larger releases are applied. Thus the mean distribution of the cross-bridges is not altered by the quick release even though the tension developed decreases drastically [15]. It seems safe to conclude that synchronization is not improved by quick release.

During contraction cross-bridges are functionally asynchronous [16]. If, at the end of the quick release, cross-bridges were really synchronized they would perform the next working stroke at the same time and the tension developed would be much larger than the isometric tension. In fact, in the whole half sarcomere, synchronization would occur, not only in series (in the ambit of the single thick filament) but also in parallel (between all the thick filaments of the half sarcomere. Moreover tension would oscillate up to the point where asynchrony would again take place.

### 3.4. The frequency of the working stroke

All the thin filaments of the half sarcomere attach to the same Z-disk therefore the force delivered by all the attached cross-bridge applies to the same Z-disk and contribute to the tension of the fibre. If this is true the frequency of the contractile events, with reference to the whole half sarcomere is given by:

$$\text{Frequency}\;\text{of}\;\text{the}\;\text{working}\;\text{strokes}={\text{N}}_{\text{F}}\cdot {\text{N}}_{\text{H}}\cdot {\text{k}}_{\text{ATPase}}$$

Where, N_F_ = 2000 – 8000 is the number of the thick filaments per sarcomere; N_H_ = 300 is the number of the myosin heads per half sarcomere; the average ATPase rate, k_ATPase_ ranges between 7.42 s^−1^ (isometric state) and 17.57 s^−1^ [7]. Thus, with reference to the whole half sarcomere, the frequency of the working strokes spans from 4.65×10^6^ s^−1^ to 42×10^6^ s^−1^ or, depending on muscle condition, one working stroke takes place every 0.21 μs or every 0.024 μs, as the average. This is not to say that the ATPase rates are accumulative. It only means that in 320 μs many working strokes take place in the half sarcomere. So the opinion of Dobie *et al*. [6] that the working stroke does not contribute to the force response during a 320 μs cycle does not appear to be justified as it is not justified the opinion that the quick release is not influenced by the working stroke. As a matter of fact the working strokes modulate continuously the state of the fibre in such a subtle way that it cannot be documented with the presently available tools. This is also the reason why the early recovery should not be taken as a measure of the working stroke.

## 4. Conclusions

The Huxley-Simmons manoeuvre was exploited to unravel the mechanics of muscle fibre contraction, in particular the regeneration of the myosin power stroke [3,4], the elastic bending and the active tilting of myosin heads [6], the quick release and the frequency of the power strokes [7,8]. In all the cases it was not taken into account that, if the rapid component of the manoeuvre is elastic as it seems to be, a fraction of the energy involved in the rapid release must be available for the rapid tension recovery. Having overlooked this fact certainly changes the overall economy of the energetic of the Huxley-Simmons manoeuvre and casts doubt on the conclusions of the literature reported in this work.

## Appendix A **(c.g.s.units)**

### Computing the rate of shortening of a stretched elastic body

The stiffness of the half sarcomere is given by, f_0_ / Δl, where f_0_ = P_0_ sez; P_0_ = 2.694×10^6^ dyne / cm^2^ is the isometric load; sez = 1.89×10^−4^ cm^2^ is the section of the fibre; and Δl = 6 nm is the stretching per half sarcomere. When referred to the whole fibre:

(A1) $$\text{stiffness}=\text{nhs}\;{\text{f}}_{0}\;/\;\Delta \text{l}$$

where, nhs, is the number of the half sarcomeres in the fibre.

The mass of the fibre is:

(A2) m=nhs d sez ls/2

where, d = 1.19 g/cm^3^, is the density of the fibre corrected for the contribution of the markers [2] and ls = 2×10^−4^ cm, is the length of the sarcomere.

The driving acceleration is:

(A3) $${\text{a}}_{\text{d}}={\text{f}}_{0}\;/\;\text{m}$$

Once these three parameters are known, computation is started, each cycle, t_A_, lasting 10^−8^ s. In each cycle the following parameters are calculated, l_A_, the shortening per cycle; s_T_, the fibre length; v, the shortening velocity; f_0_, a_d_, k_E_, the kinetic energy. The procedure is as follows:

(A4) $${\text{l}}_{\text{A}}={\text{a}}_{\text{d}}/2\;{\text{t}}_{\text{A}}^{2}+\text{v}\;{\text{t}}_{\text{A}}$$

in the first cycle, l_A_ = 0 and v = 0.

(A5) $${\text{s}}_{\text{T}}={\text{s}}_{\text{T}}\;-\;{\text{l}}_{\text{A}}$$

(A6) $$\text{v}=\text{v}+{\text{a}}_{\text{d}}\;{\text{t}}_{\text{A}}$$

(A7) $${\text{f}}_{0}=\text{stiffness}\;{\text{s}}_{\text{T}}$$

(A8) $${\text{a}}_{\text{d}}={\text{f}}_{0}/\text{m}$$

(A9) $${\text{k}}_{\text{E}}=\text{m}\;{\text{v}}^{2}/2$$

This model assumes perfect elasticity and gives rise to a harmonic motion (Figure 1 and 2). Shortening of the half sarcomere by 6 nm decreases the elastic force of the fibre to zero in the meanwhile the kinetic energy of the fibre is at the maximum.

## Appendix B **(c.g.s. units)**

### The reflection of a wave against a hard boundary

At the moment of the reflection (at the abscissa axis) both the force and the acceleration equal zero while velocity is at the maximum. The reflected wave is generated by the kinetic energy available at the moment of the impact. Each cycle of the computation lasts t_A_ = 10^−8^ s. In the first cycle a_d_ = 0 and v is the velocity at the moment of the reflection. In each cycle l_A_, s_T_, v, f_0_, a_d_ and k_E_ are calculated:

(B1) $${\text{l}}_{\text{A}}={\text{a}}_{\text{d}}\;/2\;{\text{t}}_{\text{A}}^{2}+\text{v}\;{\text{t}}_{\text{A}}$$

(B2) $${\text{s}}_{\text{T}}={\text{s}}_{\text{T}}+{\text{l}}_{\text{A}}$$

(B3) $$\text{v}=\text{v}\;-\;{\text{a}}_{\text{d}}\;{\text{t}}_{\text{A}}$$

(B4) $${\text{f}}_{0}=\text{stiffness}\;{\text{s}}_{\text{T}}$$

(B5) $${\text{a}}_{\text{d}}={\text{f}}_{0}/\text{m}$$

(B6) $${\text{k}}_{\text{E}}=\text{m}\;{\text{v}}^{2}/2$$

## Appendix C **(c.g.s.units)**

### “Isometric” rapid recovery and mimicry of the experimental rapid recovery

The operations of APPENDIX C are aimed to reproduce: 1) “Isometric” rapid recovery (Figures 4 and 5) fiber stiffness, 1.2713×10^9^ dyne/cm; mass, 0.9608 g; initial kinetic energy, 0.8159 erg; 2) the experimental rapid recovery (Figure 6) fiber stiffness, 4.767×10^6^ dyne/cm, mass, 0.32427 g; kinetic energy, 0.0097 erg, i.e. the kinetic energy available at the end of the experimental quick release of 6 nm (Figure 14 of Ford *et al*., [2]). Each cycle of the computation lasts t_A_ = 10^−8^ s. In the first cycle a_d_ = 0, f_0_ = 0, s_T_ = 0 and v = (2 k_E_/m)^0.5^ is the velocity at the moment of the reflection. In each cycle l_A_, s_T_, v, f_0_, a_d_ and k_E a_re calculated:

(C1) $$\text{la}=\text{v}\;{\text{t}}_{\text{A}}-{\text{a}}_{\text{d}}/2\;{\text{t}}_{\text{A}}^{2}$$

(C2) $${\text{s}}_{\text{T}}={\text{s}}_{\text{T}}+{\text{l}}_{\text{A}}$$

(C3) $$\text{v}=\text{v}-{\text{a}}_{\text{d}}\;{\text{t}}_{\text{A}}$$

(C4) $${\text{f}}_{0}=\text{stiffness}\;{\text{s}}_{\text{T}}$$

(C5) $${\text{a}}_{\text{d}}={\text{f}}_{0}/\text{m}$$

(C6) $${\text{k}}_{\text{E}}=\text{m}\;{\text{v}}^{2}/2$$
